# Associations of *HLA-A* and *HLA-B* with vancomycin-induced drug reaction with eosinophilia and systemic symptoms in the Han-Chinese population

**DOI:** 10.3389/fphar.2022.954596

**Published:** 2022-11-25

**Authors:** Chuang-Wei Wang, Wei-Chen Lin, Wei-Ti Chen, Chun-Bing Chen, Chun-Wei Lu, Hsin-Han Hou, Rosaline Chung-Yee Hui, Jennifer Wu, Chih-Jung Chang, Ya-Ching Chang, Wen-Hung Chung, Taiwan Severe Cutaneous Adverse Reaction Consortium

**Affiliations:** ^1^ Department of Dermatology, Drug Hypersensitivity Clinical and Research Center, Chang Gung Memorial Hospital, Linkou, Taiwan; ^2^ Cancer Vaccine and Immune Cell Therapy Core Laboratory, Chang Gung Memorial Hospital, Linkou, Taiwan; ^3^ Chang Gung Immunology Consortium, Chang Gung Memorial Hospital, Chang Gung University, Taoyuan, Taiwan; ^4^ Department of Dermatology, Xiamen Chang Gung Hospital, Xiamen, China; ^5^ College of Medicine, Chang Gung University, Taoyuan, Taiwan; ^6^ Department of Medical Education, Linkou Chang Gung Memorial Hospital, Taoyuan, Taiwan; ^7^ Whole-Genome Research Core Laboratory of Human Diseases, Chang Gung Memorial Hospital, Keelung, Taiwan; ^8^ Immune-Oncology Center of Excellence, Chang Gung Memorial Hospital, Linkou, Taiwan; ^9^ Graduate Institute of Clinical Medical Sciences, College of Medicine, Chang Gung University, Taoyuan, Taiwan; ^10^ Graduate Institute of Oral Biology, School of Dentistry, College of Medicine, National Taiwan University, Taipei, Taiwan; ^11^ Central Research Laboratory, Department of Dermatology and Xiamen Chang Gung Allergology Consortium, Xiamen Chang Gung Hospital, School of Medicine, Huaqiao University, Xiamen, China; ^12^ Department of Dermatology, Beijing Tsinghua Chang Gung Hospital, School of Clinical Medicine, Tsinghua University, Beijing, China; ^13^ Department of Dermatology, Ruijin Hospital School of Medicine, Shanghai Jiao Tong University, Shanghai, China; ^14^ Genomic Medicine Core Laboratory, Chang Gung Memorial Hospital, Linkou, Taiwan

**Keywords:** severe cutaneous adverse drug reactions, drug reaction with eosinophilia and systematic symptoms, HLA, vancomycin, delayed-type drug hypersensitivity reactions

## Abstract

Vancomycin is a commonly used antibiotic; however, it can cause life-threatening severe cutaneous adverse reactions, such as drug reaction with eosinophilia and systemic symptoms (DRESS). A previous study has reported a strong association between HLA-A*32:01 and vancomycin-induced DRESS in European ethnicity. Herein, we aim to investigate the genetic predisposition of vancomycin-induced DRESS in the Han-Chinese population. In this study, we enrolled a total of 26 patients with vancomycin-induced DRESS, 1,616 general population controls, and 51 subjects tolerant to vancomycin. *In vitro* granulysin-based lymphocyte activation tests (LAT) were conducted among 6 vancomycin-induced DRESS patients who were concomitantly receiving other medicines. *HLA-A* and *HLA-B* genotypes were determined by sequencing-based typing. Our results found that vancomycin-induced DRESS was associated with HLA-A*32:01 [odds ratio (OR) = 7.8, 95% confidence interval (CI) = 1.7–35.8; *p*-value = 0.035], HLA-B*07:05 (OR = 32.3, 95% CI = 2.8–367.7; *p*-value = 0.047), HLA-B*40:06 (OR = 4.7, 95% CI = 1.3–16.1; *p*-value = 0.036) and HLA-B*67:01 (OR = 44.8, 95% CI = 7.2–280.4; *p*-value = 0.002) when comparing the vancomycin-induced DRESS patients with the general population controls. LAT results showed that granulysin significantly increased in the vancomycin-induced DRESS patients upon vancomycin stimulation (4.7 ± 3.7 fold increased), but not upon other co-medicines. This study identified that, in addition to HLA-A*32:01, HLA-B*07:05, HLA-B*40:06, and HLA-B*67:01 were also genetic markers for vancomycin-induced DRESS in the Han-Chinese population. Associations of ethnic variances in *HLA* with vancomycin-DRESS were observed.

## Introduction

Vancomycin is a glycopeptide antibiotic that was first isolated from Amycolatopsis orientalis by Edmund Kornfeld in 1953, and it is primarily treated against Staphylococcal and Streptococcal infections, especially against those of Methicillin-resistant *Staphylococcus aureus*. While the incidence of hospital-acquired Methicillin-resistant *Staphylococcus aureus* infections has declined over the past two decades (Landrum et al., 2012; Tong et al., 2015; Rhoads et al., 2021), vancomycin remains to be widely used. Consequently, adverse effects, such as ototoxicity, nephrotoxicity (Rybak et al., 1999; Carreno et al., 2014), and vancomycin infusion reaction (Symons et al., 1985; Hepner and Castells, 2003), were reported around the globe. Aside from the above, vancomycin also causes a T-cell mediated delayed-type hypersensitivity reaction coined drug reaction with eosinophilia and systemic symptoms (DRESS) (Kwon et al., 2006; Tamagawa-Mineoka et al., 2007; Vauthey et al., 2008). DRESS is a hypersensitivity reaction consisting of early symptoms of fever, lethargy, and lymphadenopathy 2–8 weeks after the start of the treatment (Kardaun et al., 2013a). The patient later exhibits skin rash of the face, upper body, and extremities (Chen et al., 2010; Kardaun et al., 2013a). Other systemic injuries may also occur to organs, such as the liver, kidney, heart, or lungs (Chen et al., 2010; Cacoub et al., 2011; Kardaun et al., 2013a). A mortality rate of 2%–10% has been reported with the primary causes being multiple organ failure (Chiou et al., 2008; Chen et al., 2010; Cacoub et al., 2011; Kardaun et al., 2013a). In addition, long-term complications, such as autoimmune thyroiditis, lupus erythematosus, type 1 diabetes, alopecia, vitiligo, and autoimmune hemolytic anemia may inflict on patients surviving DRESS (Chen et al., 2013; Ushigome et al., 2013; Lian et al., 2018). Vancomycin is among other common antibiotics that induce DRESS besides sulfonamides (Blumenthal et al., 2012). As a result, it is of great importance that exploration of predisposition factors be warranted.

DRESS, along with the infamous Stevens-Johnson syndrome/toxic epidermal necrolysis (SJS/TEN), belongs to severe cutaneous adverse drug reactions (SCARs), which have been proven to be strongly associated with genetic human leukocyte antigen (*HLA*) alleles (Chung et al., 2004; Pan et al., 2017; Lo et al., 2020; Yang et al., 2021). For example, HLA-B*58:01 is strongly associated with allopurinol-SCARs in Han-Chinese, Japanese, Korean, Thai, and European populations (Hung et al., 2005; Kaniwa et al., 2008; Lonjou et al., 2008; Tassaneeyakul et al., 2009; Kang et al., 2011; Ng et al., 2016); HLA-A*31:01 is associated with carbamazepine-DRESS among Chinese and Europeans (Genin et al., 2014; Mockenhaupt et al., 2019), and HLA-B*13:01 is strongly associated with dapsone- and co-trimoxazole-DRESS (Zhang et al., 2013; Satapornpong et al., 2021; Wang et al., 2021).

A preceding research published by Konvinse et al. (1019) demonstrated a strong association between HLA-A*32:01 and vancomycin-induced DRESS, in which 19 (82.6%) out of 23 vancomycin-associated DRESS patients carried HLA-A*32:01. Nonetheless, this research carried out in the United States recruited patients of predominantly European ancestry, and to date, no other studies have illustrated evidence of a similar association between HLA-A*32:01 and vancomycin-induced DRESS among other ethnic groups. Within Asian populations, associations between the same or other *HLA* alleles and vancomycin-induced DRESS were not yet confirmed. Therefore, we aim to further explore the genetic predisposition of vancomycin-induced DRESS in the Han-Chinese population, and investigate whether HLA-A*32:01 or other *HLA* alleles are associated with vancomycin-induced DRESS.

## Materials and methods

### Participants

26 cases of vancomycin-associated DRESS were retrospectively enrolled from the Taiwan-SCAR consortium (including Chang Gung Memorial Hospitals, National Taiwan University Hospital, Taipei Veterans General Hospital, Taichung Veterans General Hospital, and National Cheng Kung University Hospital) in our study from 2010 to 2022. The patients’ clinical data, blood, and plasma samples were collected. Another 51 patients who had received vancomycin for at least 14 consecutive days and a total course of more than 4 weeks without evidence of adverse reactions were enrolled in the control group, and clinical data and DNA samples of whom were withdrawn. We also collected DNA samples and *HLA* genotypes of 1,616 individuals without any history of drug hypersensitivity as the general population control group, as reported previously (Wang et al., 2021). All of the subjects were of Han-Chinese ethnicity from Taiwan.

### Standard protocol approvals, registrations, and patient consent

Written informed consents were obtained from each patient of this study, and the institutional review board and ethics committee of Chang Gung Memorial Hospital have approved this study by Taiwan law (No. 97–0509B and No. 100–4657A3, 104–0291B, 201601761B0, and 201902171A3).

### Disease assessment

Every patient in our study went through assessments by at least two dermatologists. We determined the culprit drug that induced DRESS by the Naranjo algorithm and the assessment of drug causality issued by the RegiSCAR group (Naranjo et al., 1981; Sassolas et al., 2010; Kardaun et al., 2013a). Only patients met with the criteria for probable or definite cases provoked by vancomycin (Naranjo algorithm > 5) were enrolled. The consensus definition was then administered for phenotypes classification (Bastuji-Garin et al., 1993; Cacoub et al., 2011; Kardaun et al., 2013b). Clinically, the criteria and scoring system of the RegiSCAR group, which are as follows, cutaneous involvement with typical skin eruptions (e.g., exfoliative dermatitis, generalized maculopapular exanthema), fever (> 38.5 °C), enlarged lymph nodes (two or more sites, 1 cm), presence of atypical lymphocytes and eosinophilia, systemic involvement (e.g., liver, kidney, and lung), time of resolution, and the evaluation of other potential causes, were applied to diagnose DRESS. In our study, indications for vancomycin treatment, dosage and duration of vancomycin use, internal organ involvement, hematologic abnormalities, and mortality were as well examined.

### Lymphocyte activation test

Among all the cases of vancomycin-induced DRESS, 6 subjects were concomitantly receiving medicines (including amoxicillin, ceftriaxone, teicoplanin, valproic acid, diclofenac, and esomeprazole) besides vancomycin. Aside from assessment by Naranjo score, *in vitro* lymphocyte activation tests (LAT) were conducted on the 6 patients to identify whether vancomycin is the culprit drug. 5 other patients from the tolerant control group also underwent lymphocyte activation tests. We first collected PBMCs from the subjects’ whole blood samples using Ficoll-Paque (Pharmacia Fine Chemicals, Uppsala, Sweden) density gradient centrifugation. The PBMCs (1.0 × 10^6^ per well) of these subjects were subsequently cultured in 96-well microplates in RPMI-1640 medium (Gibco Invitrogen, Life Technologies, Carlsbad, CA) complemented with 10% human AB serum (Sigma-Aldrich, Darmastadt, Germany), IL-7 (Invitrogen), and vancomycin (20 ug/ml, Sigma-Aldrich, Darmastadt, Germany)/concomitant medicines that the patients were receiving respectively, and tested at 37°C in 5% CO_2_ for 1 week. Drugs were diluted in the medium to reach a concentration displaying a 10-fold physiological therapeutic level [which is a concentration of 400 ug/ml for vancomycin according to a Cmax of 42.5 ug/ml (Suzuki et al., 2012)]. Additionally, dimethyl sulfoxide was used as the solvent control and supplemented to the medium, and we used phytohemagglutinin (i.e., PHA) 10 mcg/ml as the positive control. On day 7, culture supernatants were recovered to quantify the secretions of granulysin, known to be the high specific cytotoxicity protein in DRESS patients (Weinborn et al., 2016; Su et al., 2017), by ELISA (using anti-granulysin antibodies, H3- and B04 biotin-labeled, that are produced by our laboratory). Granulysin level of 1.56 ng/ml was determined as the sensitivity cut-point of these tests. We normalized the fold change in each sample by solvent control. A positive result was defined as a 1.4-fold increase in granulysin expression in comparison with the tolerant control subjects. The cut-off value was calculated by using the values of the mean and 2-fold standard deviation from the tolerant control subjects.

#### *HLA* genotyping

*HLA-A* and *HLA-B* genotypes were decided by using SeCore HLA sequence-based typing (Invitrogen, Life Technologies, Carlsbad, CA) or *HLA* next-generation sequencing genotyping; the latter was performed by applying the Holotype HLATM X2-96/7 (no. 1056733; Omixon Biocomputing, Budapest, Hungary) on MiniSeq System (Illumina, San Diego, CA) with *HLA* Twin software (Omixon) as described in the manufacturer’s protocol. Variances in *HLA* frequencies between the patients of vancomycin-induced DRESS, the general population of Han-Chinese in Taiwan, and the tolerant control cases were analyzed.

#### Statistical analysis

All statistical analyses in this study were conducted through SPSS for Windows, version 21.0 (IBM, Armonk, NY), and Fisher exact tests were applied for comparisons of genotype frequencies between the vancomycin-induced DRESS, the tolerant control, and the general population groups. Bonferroni correction for multiple comparisons (*n* = 14 for *HLA-A* genotypes, *n* = 25 for *HLA-B* genotypes) was administered to accommodate *Pc*-values aiming to reach sufficient power to identify different phenotypes in *HLA* variances. Haldane modifications that added 0.5 to all fields to adjust possible zero counts were applied to calculate odds ratios (ORs). We exerted a two-sided test to calculate confidence intervals and *p*-values for rate ratio estimates. We determined differences to be statistically significant by *p*-values that were lower than 0.05. A significant corrected *p* (*P*c) values were *p* = 0.0036 for *HLA-A* (0.05/14) and *p* = 0.002 for *HLA-B* (0.05/25), respectively.

## Results

### Baseline demographics

The details of baseline demographics and laboratory findings are shown in Table 1. 26 patients met the inclusion criteria for vancomycin-associated DRESS and were included in our study, including 21 men and 5 women. The average age of the subjects was 56.9 ± 19.3 years old. All patients were probable or definite cases of vancomycin-induced DRESS, with Naranjo algorithm > 5. The mean received dosage of vancomycin was 1,628.8 mg/day ± 584.2 mg/day and the duration to DRESS onset after the first day of vancomycin treatment was from 14 to 62 days (the average was 20.6 ± 12.9 days). 6 out of 26 subjects were concomitantly receiving other medicines (including amoxicillin, ceftriaxone, teicoplanin, valproic acid, diclofenac, and esomeprazole) when prescribed with vancomycin.

**TABLE 1:** Demographic and baseline clinical characteristics of vancomycin-induced DRESS and tolerant controls.

Characteristics	Vancomycin-induced Dress *N* = 26	Vancomycin tolerant controls *N* = 51	p-Value
Age, years, mean ± SD	56.9 ± 19.3	55.5 ± 16.6	0.753*
Sex, *n* (%)			0.417
Male	21 (80.8%)	36 (70.6%)	
Female	5 (19.2%)	15 (29.4%)	
Deceased cases, No. (%)	2 (7.7%)	0 (0%)	0.111
Internal organ involvement			
Hepatitis, GPT, IU/L, No. (%)			< 0.001
Normal^a^	13 (50.0%)	49 (96.1%)	
> 3 fold	13 (50.0%)	2 (3.9%)	
Acute renal failure^b^	10 (38.5%)	2 (3.9%)	<0.001
Hematologic abnormalities			
Eosinophilia, absolute eosinophil count		n.d	
< 500/μl	9 (34.6%)	n.d	
≥ 500/μl	17 (65.4%)	n.d	
Atypical lymphocytosis	7 (26.9%)	n.d	n.d
Vancomycin exposure			
Dosage, mean ± SD	1,628.8 ± 584.2	1,582.3 ± 521.0	0.723*
[range], mg/d	[250–2,400]	[500–2000]	
Duration to DRESS onset/Treatment duration, mean ± SD	20.6 ± 12.9^&^	> 14^#^	n.d
[range], d			
Indication of Vancomycin, No. (%)			0.894^@^
Pneumonia	4 (15.4%)	10 (19.6%)	
Unknown fever/infection	6 (23.1%)	10 (19.6%)	
Cellulitis	5 (19.2%)	9 (17.6%)	
CNS infection	3 (11.5%)	5 (9.8%)	
Infectious endocarditis	3 (11.5%)	5 (9.8%)	
Sepsis	2 (7.7%)	6 (11.8%)	
Wound infection	2 (7.7%)	6 (11.8%)	
Acute pancreatitis	1 (3.8%)	0 (0%)	
Underlying diseases, No. (%)			0.915^@^
Hypertension	8 (30.8%)	19 (37.3%)	
CKD	3 (11.5%)	8 (15.7%)	
CVD	4 (15.4%)	6 (11.8%)	
DM	6 (23.1%)	10 (19.6%)	
Malignant cancer	4 (15.4%)	5 (9.8%)	
CNS diseases	4 (15.4%)	5 (9.8%)	

*p* values were calculated by using Fisher’s exact test, *These p values were calculated by using Student *t*-test^, @^These p values were calculated by using Chi-Squared test, ^&^The duration to DRESS onset of vancomycin-induced DRESS patients after the first day of vancomycin treatmnt was from 14 to 62 days (the average was 20.6 ± 12.9 days), #51 subjects who had received vancomycin for at least 14 consecutive days and a total course of more than 4 weeks without evidence of adverse reactions were enrolled as the controls. Abbreviations: DRESS, drug reaction with eosinophilia and systemic symptoms; CKD, chronic kidney disease; CNS, central nervous system; CVD, cardiovascular disease; DM, diabetes mellitus; GPT, glutamic pyruvic transaminase; n.d, not determined.

^a^ 36 U/L according to the lab’s reference.

^b^ An elevation of serum creatinine value greater than 1.5-fold of the normal value range (0.4 mg/dl–1.5 mg/dl) after drug intake.

51 subjects who had received vancomycin for at least 14 consecutive days and a total course of more than 4 weeks without evidence of adverse reactions were enrolled in the control group, including 36 males and 15 females. The average age of tolerant control cases was 55.5 ± 16.6 years old. The mean dosage received was 1,582.3 mg ± 521.0 mg daily. There were no significant differences in age and the exposed vancomycin dosage between the vancomycin-induced DRESS and the tolerant control groups.

### Laboratory findings

Of all patients, elevated GPT serum level up to 3 folds of normal upper limit (that is 36 U/L according to the lab’s reference) was found in 13 subjects (50.0%), and 10 subjects (38.5%) presented with acute renal failure [defined as an elevation of serum creatinine level greater than 1.5 folds of the normal value range (0.4 mg/dl–1.5 mg/dl) after drug intake]. 17 patients (65.4%) showed eosinophilia (absolute eosinophil count > 500/μl) while 7 cases (26.9%) exhibited atypical lymphocytosis. There was a total of 2 deceased cases out of the 26 patients with vancomycin-induced DRESS (7.7%).

### Lymphocyte activation test results

**FIGURE 1:**
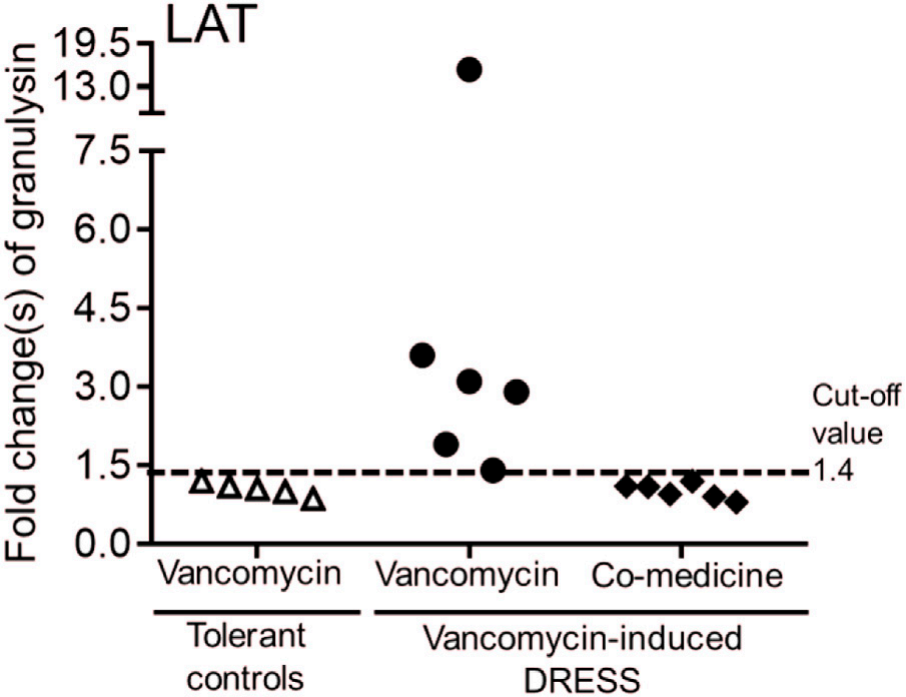
Lymphocyte activation test (LAT) for patients with vancomycin-induced DRESS. Granulysin-based lymphocyte activation test (LAT) was performed in 6 patients with vancomycin-DRESS and 5 tolerant controls. A positive result was defined as a 1.4-fold increase in granulysin release compared to the tolerant controls (dotted line).

6 out of 26 subjects were concomitantly receiving other medicines (such as amoxicillin, ceftriaxone, teicoplanin, valproic acid, diclofenac, and esomeprazole) when prescribed with vancomycin. We performed *in vitro* granulysin-based lymphocyte activation tests (LAT) (Lin et al., 2018; Chu et al., 2021) to further determine the culprit drug of these DRESS patients. LAT assay was also conducted on 5 subjects from the tolerant control group. We determined the cut-off values to be a 1.4-fold increase in granulysin expression. LAT results showed that granulysin expression (4.7 ± 3.7 fold increased) of these 6 subjects with DRESS all exceeded the cut-off value when the PBMCs of these cases were cultured with vancomycin for 1 week (Figure 1). On the other hand, when the subjects’ PBMCs were cultured in the presence of the concomitantly-received medicines, no increase over the cut-off value in granulysin expression was noticed (Figure 1), suggesting that all these 6 DRESS cases were vancomycin-induced.

### Association of HLA-A*32:01, HLA-B*07:05, HLA-B*40:06, and HLA-B*67*01 of patients with vancomycin-induced DRESS

The details of *HLA-A* and *HLA-B* genotypes in the patients with vancomycin-induced DRESS are shown in Table 2. All subjects were from Taiwan and of Han-Chinese ethnicity. We first compared the 26 vancomycin-induced DRESS patients with 1,616 general population controls from Taiwan, the results found associations between HLA-A*32:01, HLA-B*07:05, HLA-B*40:06 and HLA-B*67:01, and vancomycin-induced DRESS (Table 3). HLA-A*32:01 was present in 7.7% (2/26) of the vancomycin-associated DRESS patients, and only in 1.1% (17/1,616) of the general Han-Chinese population (odds ratio [OR] = 7.8, 95% confidence interval [CI] = 1.7–35.8; *p* = 0.035; sensitivity = 7.7%, specificity = 98.9%). HLA-B*07:05 was present in 3.8% (1/26) of the vancomycin-associated DRESS cases, and in 0.1% (2/1,616) of the general Han-Chinese population (OR = 32.3, 95% CI = 2.8–367.7; *p* = 0.047; sensitivity = 3.8%, specificity = 99.9%). HLA-B*40:06 was present in 11.5% (3/26) of the vancomycin-associated DRESS cases, and in 2.7% (44/1,616) of the general Han-Chinese population (OR = 4.7, 95% CI = 1.3–16.1; *p* = 0.036; sensitivity = 11.5%, specificity = 99.1%). HLA-B*67:01 was present in 7.7% (2/26) of the vancomycin-associated DRESS patients, and in 0.2% (3/1,616) of the general Han-Chinese population (OR = 44.8, 95% CI = 7.2–280.4; *p* = 0.002; sensitivity = 7.7%, specificity = 99.8%) (Tables 3, 4). We then compared the vancomycin-induced DRESS patients with the 51 tolerant controls and discovered that HLA-A*32:01 (OR = 10.5, 95% CI = 0.5–227; *p* = 0.111), HLA-B*07:05 (OR = 6.1, 95% CI = 0.2–154; *p* = 0.338), HLA-B*40:06 (OR = 3.2, 95% CI = 0.5–20.5; *p* = 0.329) and HLA-B*67:01 (OR = 10.5, 95% CI = 0.5–227; *p* = 0.111) also imposed risks of vancomycin-induced DRESS (Table 4); however, these associations were insignificant as calculated *P*c values were higher than the 0.05 for statistical significance for *HLA-A* and *HLA-B* respectively. Further analyses were performed and came upon stronger associations between the combined four alleles of HLA-A*32:01, HLA-B*07:05, HLA-B*40:06, and HLA-B*67:01 and vancomycin-induced DRESS when we compared the vancomycin-induced DRESS patients with both the general population group (OR = 7.7, 95% CI = 3.0–19.8; *p* = 4.8 × 10^−4^; sensitivity = 23.1%, specificity = 96.2%) and the tolerant control cases (OR = 7.4, 95% CI = 1.4–39.5; *p* = 0.016) (Table 4). Positive and negative predictive values of the four respective genetic factor and the combined genetic factor were not calculated on account of lack of current liable data on incidence of vancomycin-induced DRESS.

**TABLE 2:** The detailed genotypes of HLA-A and HLA-B in patients with vancomycin-induced DRESS.

DRESS Case	Phenotype	RegiSCAR scoring for DRESS*	HLA			
			HLA-A		HLA-B	
1	DRESS	4	A*02:01	A*33:03	B*48:01	B*58:01
2	DRESS	5	A*02:03	A*24:02	B*40:01	B*40:01
3	DRESS	6	A*11:01	A*33:03	B*40:01	B*58:01
4	DRESS	5	A*24:02	A*24:02	B*15:25	B*40:01
5	DRESS	5	A*24:02	A*33:03	B*40:06	B*58:01
6	DRESS	4	A*26:01	A*32:01	B*44:03	B*67:01
7	DRESS	4	A*02:07	A*11:01	B*40:01	B*46:01
8	DRESS	4	A*11:01	A*33:03	B*08:01	B*58:01
9	DRESS	5	A*02:01	A*24:02	B*40:06	B*67:01
10	DRESS	4	A*02:01	A*02:07	B*13:01	B*40:01
11	DRESS	4	A*11:01	A*11:01	B*13:01	B*15:02
12	DRESS	4	A*24:02	A*26:01	B*46:01	B*55:02
13	DRESS	4	A*02:06	A*11:01	B*48:01	B*51:02
14	DRESS	5	A*11:01	A*11:01	B*13:01	B*46:01
15	DRESS	4	A*02:07	A*11:02	B*27:04	B*46:01
16	DRESS	4	A*24:02	A*30:01	B*13:02	B*58:01
17	DRESS	4	A*11:01	A*32:01	B*40:01	B*44:03
18	DRESS	6	A*02:01	A*33:03	B*13:01	B*13:01
19	DRESS	4	A*02:01	A*11:02	B*40:01	B*46:01
20	DRESS	4	A*02:03	A*11:01	B*38:02	B*46:01
21	DRESS	4	A*02:07	A*11:01	B*13:01	B*46:01
22	DRESS	4	A*02:03	A*11:01	B*38:02	B*40:01
23	DRESS	4	A*11:01	A*24:02	B*07:05	B*40:01
24	DRESS	6	A*02:06	A*11:01	B*40:06	B*58:01
25	DRESS	4	A*11:01	A*24:02	B*46:01	B*51:01
26	DRESS	4	A*11:01	A*33:03	B*51:01	B*58:01

*RegiSCAR, Scoring for DRESS, was based on the criteria and scoring system of the RegiSCAR, group (Cacoub et al., 2011) (Kardaun et al., 2013b). Abbreviations: DRESS, drug reaction with eosinophilia and systemic symptoms; HLA, human leukocyte antigen. HLA genes were written in italics as standard formatting.

**TABLE 3:** The associations study of HLA-A and HLA-B alleles in patients with vancomycin-induced DRESS compared to general population controls.

HLA genotype	Vancomycin- DRESS, *N* = 26 N (%)	General population control, *N* = 1,616 N (%)	OR (95% CI)	*p* Values
A*02:01	5 (19.2%)	328 (20.3%)	0.9 (0.3–2.5)	1.000
A*02:03	3 (11.5%)	202 (12.5%)	0.9 (0.3–3.1)	1.000
A*02:06	2 (7.7%)	97 (6.0%)	1.3 (0.3–5.6)	0.668
A*02:07	4 (15.4%)	267 (16.5%)	0.9 (0.3–2.7)	1.000
A*11:01	14 (53.8%)	797 (49.3%)	1.2 (0.6–2.6)	0.696
A*11:02	2 (7.7%)	133 (8.2%)	0.9 (0.2–4.0)	1.000
A*24:02	8 (30.8%)	487 (30.1%)	1.0 (0.4–2.4)	1.000
A*26:01	2 (7.7%)	90 (5.6%)	1.4 (0.3–6.1)	0.654
A*30:01	1 (3.8%)	58 (3.6%)	1.1 (0.1–8.1)	0.617
A*32:01	2 (7.7%)	17 (1.1%)	7.8 (1.7∼35.8)	0.035
A*33:03	6 (23.1%)	369 (22.8%)	1.0 (0.4–2.5)	1.000
B*07:05	1 (3.8%)	2 (0.1%)	32.3 (2.8∼367.7)	0.047
B*08:01	1 (3.8%)	8 (0.5%)	8.0 (1.0–66.7)	0.134
B*13:01	5 (19.2%)	186 (11.5%)	1.8 (0.7–4.9)	0.216
B*13:02	1 (3.8%)	59 (3.7%)	1.1 (0.1–7.9)	1.000
B*15:02	1 (3.8%)	133 (8.2%)	0.4 (0.1–3.3)	0.717
B*15:25	1 (3.8%)	18 (1.1%)	3.6 (0.5–27.6)	0.263
B*27:04	1 (3.8%)	89 (5.5%)	0.7 (0.1–5.1)	1.000
B*38:02	2 (7.7%)	122 (7.5%)	1.0 (0.2–4.4)	1.000
B*40:01	9 (34.6%)	583 (36.1%)	0.9 (0.4–2.1)	1.000
B*40:06	3 (11.5%)	44 (2.7%)	4.7 (1.3∼16.1)	0.036
B*44:03	2 (7.7%)	26 (1.6%)	5.1 (1.1–22.7)	0.071
B*46:01	8 (30.8%)	366 (22.6%)	1.5 (0.7–3.5)	0.346
B*48:01	2 (7.7%)	36 (2.2%)	3.7 (0.8–16.1)	0.120
B*51:01	2 (7.7%)	135 (8.4%)	0.9 (0.2–3.9)	1.000
B*51:02	1 (3.8%)	51 (3.2%)	1.2 (0.2–9.2)	0.570
B*55:02	1 (3.8%)	90 (5.6%)	0.7 (0.1–5.1)	1.000
B*58:01	7 (26.9%)	331 (20.5%)	1.4 (0.6–3.4)	0.462
B*67:01	2 (7.7%)	3 (0.2%)	44.8 (7.2∼280.4)	0.002

Abbreviations: 95% CI, 95% Confidence Interval; DRESS, drug reaction with eosinophilia and systemic symptoms; HLA, human leukocyte antigen; OR, odds ratio. p values were calculated by Fisher’s exact test.

**TABLE 4:** Association of HLA-A*32:01, HLA-B*07:05, HLA-B*40:06, and HLA-B*67:01 with patients with vancomycin-induced DRESS.

HLA genotypes	DRESS Case, *N* (%)	General population Ctrl, *N* (%)	OR* (95% CI)*	*p*-value*	Tolerant Ctrl, N (%)	OR^#^ (95% CI)^#^	*p*-value^#^
A*32:01	2/26 (7.7%)	17/1,616 (1.1%)	7.8 (1.7–35.8)	0.035	0/51 (0%)	10.5 (0.5–227)	0.111
B*07:05	1/26 (3.8%)	2/1,616 (0.1%)	32.3 (2.8–367.7)	0.047	0/51 (0%)	6.1 (0.2–154)	0.338
B*40:06	3/26 (11.5%)	44/1,616 (2.7%)	4.7 (1.3–16.1)	0.036	2/51 (3.9%)	3.2 (0.5–20.5)	0.329
B*67:01	2/26 (7.7%)	3/1,616 (0.2%)	44.8 (7.2–280.4)	0.002	0/51 (0%)	10.5 (0.5–227)	0.111
Combined A*32:01, B*07:05, B*40:06, and B*67:01							
	6/26 (23.1%)	62/1,616 (3.8%)	7.7 (3.0–19.8)	4.8 × 10–4	2/51 (3.9%)	7.4 (1.4–39.5)	0.016

*Data obtained from comparison of cases with the general population from Taiwan, #Data obtained from comparison of cases with relevant tolerant controls, The main ethnicity of the enrolled cases and controls was the Han-Chinese population, *p* values were calculated by Fisher’s exact test.

Based on the Allele Frequency Net Database (http://www.allelefrequencies.net/), the frequency of the HLA-A*32:01 is higher in European (3.2%–13.8%), American (3.0%–7.4%) and Indian (5.6%), but much lower in Chinese and Japanese (0%–2.1%). This may explain why HLA-A*32:01 is strongly associated with vancomycin-induced DRESS in European ethnicity (sensitivity = 82.6%, *p* = 2 × 10^−16^) (Konvinse et al., 2019), but is weakly associated in the Chinese population (sensitivity = 7.7%, *p* = 0.035, according to this study).

## Discussion

DRESS, along with SJS/TEN, is a type of life-threatening SCARs. Current comprehension of the pathogenesis regarding DRESS involves genetic polymorphism in *HLA*. Several present studies have proved genetic polymorphism in *HLA* to be of significance concerning SCARs. Allopurinol-SCARs and HLA-B*58:01 (Hung et al., 2005; Kaniwa et al., 2008; Lonjou et al., 2008; Tassaneeyakul et al., 2009; Kang et al., 2011), carbamazepine-DRESS and HLA-A*31:01 (Genin et al., 2014), and dapsone-/co-trimoxazole-DRESS and HLA-B*13:01 (Zhang et al., 2013; Satapornpong et al., 2021; Wang et al., 2021) are just a few among other identified connections. Additionally, these associations varied from different ethnic groups, and the associations are phenotype- and ethnic-specific. For instance, an association was recognized between co-trimoxazole-induced DRESS and HLA-B*13:01 in the Chinese population (Wang et al., 2021), while the same *HLA* allele was weakly associated with co-trimoxazole-induced SJS/TEN (Kongpan et al., 2015; Wang et al., 2021). Furthermore, HLA-B*15:02 was reported to be strongly associated with carbamazepine-induced SJS/TEN in Asian populations (Chung et al., 2004; Locharernkul et al., 2008; Tassaneeyakul et al., 2010; Cheung et al., 2013; Tangamornsuksan et al., 2013; Chung et al., 2016), whereas it is HLA-B*57:01 that was identified to be related to SJS/TEN among Europeans (Mockenhaupt et al., 2019). And HLA-A*31:01 was associated with carbamazepine-induced DRESS (Genin et al., 2014; Wang et al., 2022).

A strong association between HLA-A*32:01 and vancomycin-induced DRESS in patients of European ancestry has been noticed in a previous study (Konvinse et al., 2019). In our study, associations between HLA-A*32:01, HLA-B*07:05, HLA-B*40:06, and HLA-B*67:01 and vancomycin-induced DRESS in the Han-Chinese population from Taiwan were identified. These are the first documented *HLA* alleles that contribute to vancomycin-induced drug hypersensitivity among the Han-Chinese population. Although the same association between HLA-A*32:01 and vancomycin-induced DRESS was formerly observed in the previous study of Konvinse KC et al., the cases were predominantly of European, not Chinese, ancestry. And this is also the first study that recognized the associations of HLA-B*07:05, HLA-B*40:06 and HLA-B*67:01 and vancomycin-induced DRESS. No other studies to date have explored the role that polymorphism in *HLA* genotypes plays in phenotypes regarding vancomycin hypersensitivity reactions within other ethnic groups aside from the European population, specifically among the Han-Chinese population. Our study further validates the present understanding that associations between *HLA* alleles and vancomycin-DRESS differ among different ethnicities.

The execution of regular screening for HLA-B*57:01 before prescribing abacavir has greatly reduced the risk of hypersensitivity reactions in patients receiving the drug (Mallal et al., 2008). Other implementations of pharmacogenomics into clinical practice include screening for HLA-B*58:01 allele in high-risk patients before prescribing allopurinol (Ko et al., 2015). United States FDA also suggests testing for HLA-B*15:02 allele before using carbamazepine in patients of Asian ancestry (Chen et al., 2011; Wang et al., 2022). Konvinse KC et al. have proposed a protocol that advocates the importance of screening for HLA-A*32:01 in patients of European ethnicity receiving vancomycin to reduce the incidence of vancomycin-induced DRESS, and further modify their antibiotic regimen. Alternatively, our study has discovered the connections between HLA-A*32:01, HLA-B*07:05, HLA-B*40:06, and HLA-B*67:01, and a particular clinical phenotype in vancomycin-related hypersensitivity reactions in the Han-Chinese population from Taiwan. However, due to the low sensitivity (23.1%) of detection of the four combined *HLA* alleles, the clinical application of genetic HLA-A*32:01, HLA-B*07:05, HLA-B*40:06, and HLA-B*67:01 testing prior to vancomycin use seems not to be cost-effective. Nonetheless, owing to strong associations between HLA-A*32:01, HLA-B*07:05, HLA-B*40:06, and HLA-B*67:01, and vancomycin-associated DRESS, these alleles act as genetic markers for vancomycin-associated DRESS. And together with the assistance of LAT assay, the detection of HLA-A*32:01, HLA-B*07:05, HLA-B*40:06, and HLA-B*67:01 alleles may still play a vital part in the clinical decision-making process. In the past few decades, technology in the fields of gene sequencing has developed rapidly. With novel techniques, such as whole exome sequencing (WES) and whole genome sequencing (WGS), physicians can now obtain more comprehensive genetic information regarding specific diseases and personalized medicine (Dunn et al., 2018; Zhao et al., 2019). The progress in sequencing techniques allows a more encompassing pharmacogenetics profiling. The potential application of WES and/or WGS for HLA-A*32:01, HLA-B*07:05, HLA-B*40:06, and HLA-B*67:01 alleles detection may thus enable more delicate and precise selection of medication for individuals.

Aside from *HLA* genotypes, currently recognized factors in the pathogenesis of SCARs included divergence in individual drug metabolism (Chung et al., 2014; Pan et al., 2017; Lo et al., 2020), cytotoxicity mechanisms (Chung et al., 2008; Wang et al., 2013; Kuijper et al., 2020), and viral infections (Tohyama et al., 2007; Miyagawa and Asada, 2021). A study by our group formerly discovered a strong correlation between phenytoin-SCARs and CYP2C9*3, which in turn decreases phenytoin clearance (Chung et al., 2014). The discovery shed light on the role that divergence in drug metabolism plays in SCARs. Besides, drug-specific T cell receptors (TCR) also play a vital part in the pathogenesis of SCARs (Chung et al., 2015; Pan et al., 2019). For instance, the TCRβ CDR3 clonotype, “ASSLAGELF”, which showed significant carbamazepine-specific cytotoxicity, was discovered in patients with carbamazepine-SJS/TEN. *In vitro* expansion and granulysin release activation of carbamazepine-specific CD8^+^ T cells were observed on carbamazepine stimulation (Pan et al., 2019; Chu et al., 2021). Direct activation of drug-specific T cells by oxypurinol through the pharmacological interaction (p-i) mechanism was also identified by several studies (Yun et al., 2013; Yun et al., 2014; Chung et al., 2015). Therefore, other factors regarding the pathogenesis of DRESS require further exploration.

There are several limitations in this study. First, a relatively small sample size of mere 26 cases was included on account of the low incidence of DRESS. Secondly, the vancomycin treatment duration of the vancomycin-induced DRESS patients enrolled was from 14 to up to 62 days, whereas subjects were included as the tolerant control group provided at least 14 consecutive days and a total of more than 4 weeks of vancomycin treatment. Tolerant subjects that continued vancomycin use over 60 days were hard to recruit owing to few clinical scenarios that required prolonged vancomycin treatment. Finally, we did not recruit vancomycin-related SJS/TEN patients in our study; thus, the associations between divergence in *HLA* alleles and vancomycin-SJS/TEN remain unclear.

All in all, our study discovered before-unknown associations between *HLA* genotypes and vancomycin-related hypersensitivity reactions within the Han-Chinese population. Though the association between HLA-A*32:01 and vancomycin-induced DRESS was formerly observed in the previous study of Konvinse KC et al., we further identified that HLA-B*07:05, HLA-B*40:06, and HLA-B*67:01 were associated with vancomycin-induced DRESS in the Han-Chinese population. The associations of HLA-A*32:01, HLA-B*07:05, HLA-B*40:06, and HLA-B*67:01 alleles with vancomycin-induced DRESS were the first to be discovered within the Asian population. The ethnic variances in *HLA* associations with vancomycin-DRESS were observed. In addition to researches concerning *HLA* alleles, further studies on the relations of other genetic factors (such as drug metabolizing enzyme and TCR) and vancomycin-induced SCARs are required to better comprehend the pathogenesis of delayed-type hypersensitivity reactions induced by vancomycin.

## Data Availability

The datasets presented in this study can be found in online repositories. The names of the repository/repositories and accession number(s) can be found in the article/Supplementary Material.

## References

[B1] Bastuji-Garin S. Rzany B. Stern R. S. Shear N. H. Naldi L. Roujeau J. C. (1993). Clinical classification of cases of toxic epidermal necrolysis, stevens-johnson syndrome, and erythema multiforme. Archives Dermatology 129 (1), 92–96. 10.1001/archderm.129.1.92 8420497

[B2] Blumenthal K. G. Patil S. U. Long A. A. (2012). The importance of vancomycin in drug rash with eosinophilia and systemic symptoms (DRESS) syndrome. Allergy Asthma Proc. 33 (2), 165–171. 10.2500/aap.2012.33.3498 22525393

[B3] Cacoub P. Musette P. Descamps V. Meyer O. Speirs C. Finzi L. (2011). The DRESS syndrome: A literature review. Am. J. Med. 124 (7), 588–597. 10.1016/j.amjmed.2011.01.017 21592453

[B4] Carreno J. J. Kenney R. M. Lomaestro B. (2014). Vancomycin-associated renal dysfunction: Where are we now? Pharmacotherapy 34 (12), 1259–1268. 10.1002/phar.1488 25220436

[B5] Chen P. Lin J. J. Lu C. S. Ong C. T. Hsieh P. F. Yang C. C. (2011). Carbamazepine-induced toxic effects and HLA-B*1502 screening in Taiwan. N. Engl. J. Med. 364 (12), 1126–1133. 10.1056/NEJMoa1009717 21428768

[B6] Chen Y. C. Chang C. Y. Cho Y. T. Chiu H. C. Chu C. Y. (2013). Long-term sequelae of drug reaction with eosinophilia and systemic symptoms: A retrospective cohort study from taiwan. J. Am. Acad. Dermatol. 68 (3), 459–465. 10.1016/j.jaad.2012.08.009 22959230

[B7] Chen Y. C. Chiu H. C. Chu C. Y. (2010). Drug reaction with eosinophilia and systemic symptoms: A retrospective study of 60 cases. Arch. Dermatol. 146 (12), 1373–1379. 10.1001/archdermatol.2010.198 20713773

[B8] Cheung Y. K. Cheng S. H. Chan E. J. M. Lo S. V. Ng M. H. L. Kwan P. (2013). HLA-B alleles associated with severe cutaneous reactions to antiepileptic drugs in Han Chinese. Epilepsia 54 (7), 1307–1314. 10.1111/epi.12217 23692434

[B9] Chiou C. C. Yang L. C. Hung S. I. Chang Y. C. Kuo T. T. Ho H. C. (2008). Clinicopathological features and prognosis of drug rash with eosinophilia and systemic symptoms: A study of 30 cases in taiwan. J. Eur. Acad. Dermatol. Venereol. 22 (9), 1044–1049. 10.1111/j.1468-3083.2008.02585.x 18627428

[B10] Chu M. T. Wang C. W. Chang W. C. Chen C. B. Chung W. H. Hung S. I. (2021). Granulysin-based lymphocyte activation test for evaluating drug causality in antiepileptics-induced severe cutaneous adverse reactions. J. Invest. Dermatol. 141 (6), 1461–1472 e10. 10.1016/j.jid.2020.11.027 33340500

[B11] Chung W. H. Chang W. C. Lee Y. S. Wu Y. Y. Yang C. H. Ho H. C. (2014). Genetic variants associated with phenytoin-related severe cutaneous adverse reactions. JAMA 312 (5), 525–534. 10.1001/jama.2014.7859 25096692

[B12] Chung W. H. Hung S. I. Hong H. S. Hsih M. S. Yang L. C. Ho H. C. (2004). Medical genetics: A marker for stevens-johnson syndrome. Nature 428 (6982), 486. 10.1038/428486a 15057820

[B13] Chung W. H. Hung S. I. Yang J. Y. Su S. C. Huang S. P. Wei C. Y. (2008). Granulysin is a key mediator for disseminated keratinocyte death in Stevens-Johnson syndrome and toxic epidermal necrolysis. Nat. Med. 14 (12), 1343–1350. 10.1038/nm.1884 19029983

[B14] Chung W. H. Pan R. Y. Chu M. T. Chin S. W. Huang Y. L. Wang W. C. (2015). Oxypurinol-specific T cells possess preferential TCR clonotypes and express granulysin in allopurinol-induced severe cutaneous adverse reactions. J. Invest. Dermatol. 135 (9), 2237–2248. 10.1038/jid.2015.165 25946710

[B15] Chung W. H. Wang C. W. Dao R. L. (2016). Severe cutaneous adverse drug reactions. J. Dermatol. 43 (7), 758–766. 10.1111/1346-8138.13430 27154258

[B16] Dunn P. Albury C. L. Maksemous N. Benton M. C. Sutherland H. G. Smith R. A. (2018). Next generation sequencing methods for diagnosis of epilepsy syndromes. Front. Genet. 9, 20. 10.3389/fgene.2018.00020 29467791 PMC5808353

[B17] Genin E. Chen D. P. Hung S. I. Sekula P. SchuMacherM. Chang P. Y. (2014). HLA-A*31:01 and different types of carbamazepine-induced severe cutaneous adverse reactions: An international study and meta-analysis. Pharmacogenomics J. 14 (3), 281–288. 10.1038/tpj.2013.40 24322785

[B18] Hepner D. L. Castells M. C. (2003). Anaphylaxis during the perioperative period. Anesth. Analg. 97 (5), 1381–1395. 10.1213/01.ane.0000082993.84883.7d 14570656

[B19] Hung S. I. Chung W. H. Liou L. B. Chu C. C. Lin M. Huang H. P. (2005). HLA-B*5801 allele as a genetic marker for severe cutaneous adverse reactions caused by allopurinol. Proc. Natl. Acad. Sci. U. S. A. 102 (11), 4134–4139. 10.1073/pnas.0409500102 15743917 PMC554812

[B20] Kang H. R. Jee Y. K. Kim Y. S. Lee C. H. Jung J. W. Kim S. H. (2011). Positive and negative associations of HLA class I alleles with allopurinol-induced SCARs in Koreans. Pharmacogenet. Genomics 21 (5), 303–307. 10.1097/FPC.0b013e32834282b8 21301380

[B21] Kaniwa N. Saito Y. Aihara M. Matsunaga K. Tohkin M. Kurose K. (2008). HLA-B locus in Japanese patients with anti-epileptics and allopurinol-related Stevens-Johnson syndrome and toxic epidermal necrolysis. Pharmacogenomics 9 (11), 1617–1622. 10.2217/14622416.9.11.1617 19018717

[B22] Kardaun S. H. Sekula P. VaLeyrie-Allanore L. Liss Y. Chu C. Y. Creamer D. (2013). Drug reaction with eosinophilia and systemic symptoms (DRESS): An original multisystem adverse drug reaction. Results from the prospective RegiSCAR study. Br. J. Dermatol. 169 (5), 1071–1080. 10.1111/bjd.12501 23855313

[B23] Kardaun S. H. Sekula P. VaLeyrie-Allanore L. Liss Y. Chu C. Y. Creamer D. (2013). Drug reaction with eosinophilia and systemic symptoms (DRESS): An original multisystem adverse drug reaction. Results from the prospective RegiSCAR study. Br. J. Dermatol. 169 (5), 1071–1080. 10.1111/bjd.12501 23855313

[B24] Ko T. M. Tsai C. Y. Chen S. Y. Chen K. S. Yu K. H. Chu C. S. (2015). Use of HLA-B*58:01 genotyping to prevent allopurinol induced severe cutaneous adverse reactions in taiwan: National prospective cohort study. Bmj 351, h4848. 10.1136/bmj.h4848 26399967 PMC4579807

[B25] Kongpan T. Mahasirimongkol S. Konyoung P. Kanjanawart S. Chumworathayi P. Wichukchinda N. (2015). Candidate HLA genes for prediction of co-trimoxazole-induced severe cutaneous reactions. Pharmacogenet. Genomics 25 (8), 402–411. 10.1097/FPC.0000000000000153 26086150

[B26] Konvinse K. C. Trubiano J. A. Pavlos R. James I. Shaffer C. M. Bejan C. A. (2019). HLA-A*32:01 is strongly associated with vancomycin-induced drug reaction with eosinophilia and systemic symptoms. J. Allergy Clin. Immunol. 144 (1), 183–192. 10.1016/j.jaci.2019.01.045 30776417 PMC6612297

[B27] Kuijper E. French L. E. Tensen C. P. Vermeer M. H. Bouwes Bavinck J. N. (2020). Clinical and pathogenic aspects of the severe cutaneous adverse reaction epidermal necrolysis (EN). J. Eur. Acad. Dermatol. Venereol. 34 (9), 1957–1971. 10.1111/jdv.16339 32415695 PMC7496676

[B28] Kwon H. S. Chang Y. S. Jeong Y. Y. Lee S. M. Song W. J. Kim H. B. (2006). A case of hypersensitivity syndrome to both vancomycin and teicoplanin. J. Korean Med. Sci. 21 (6), 1108–1110. 10.3346/jkms.2006.21.6.1108 17179696 PMC2721938

[B29] Landrum M. L. Neumann C. Cook C. Chukwuma U. Ellis M. W. Hospenthal D. R. (2012). Epidemiology of *Staphylococcus aureus* blood and skin and soft tissue infections in the US military health system, 2005-2010. Jama 308 (1), 50–59. 10.1001/jama.2012.7139 22760291

[B30] Lian B. S. Busmanis I. Lee H. Y. (2018). Relapsing course of sulfasalazine-induced drug reaction with eosinophilia and systemic symptoms (DRESS) complicated by alopecia universalis and vitiligo. Ann. Acad. Med. Singap. 47 (11), 492–493. 10.47102/annals-acadmedsg.v47n11p492 30578426

[B31] Lin C. Y. Wang C. W. Hui C. Y. R. Chang Y. C. Yang C. H. Cheng C. Y. (2018). Delayed-type hypersensitivity reactions induced by proton pump inhibitors: A clinical and *in vitro* T-cell reactivity study. Allergy 73 (1), 221–229. 10.1111/all.13235 28658503

[B32] Lo C. Nguyen S. Yang C. Witt L. Wen A. Liao T. V. (2020). Pharmacogenomics in asian subpopulations and impacts on commonly prescribed medications. Clin. Transl. Sci. 13 (5), 861–870. 10.1111/cts.12771 32100936 PMC7485947

[B33] Locharernkul C. Loplumlert J. Limotai C. Korkij W. Desudchit T. Tongkobpetch S. (2008). Carbamazepine and phenytoin induced Stevens-Johnson syndrome is associated with HLA-B*1502 allele in Thai population. Epilepsia 49 (12), 2087–2091. 10.1111/j.1528-1167.2008.01719.x 18637831

[B34] Lonjou C. Borot N. Sekula P. Ledger N. Thomas L. Halevy S. (2008). A European study of HLA-B in Stevens-Johnson syndrome and toxic epidermal necrolysis related to five high-risk drugs. Pharmacogenet. Genomics 18 (2), 99–107. 10.1097/FPC.0b013e3282f3ef9c 18192896

[B35] Mallal S. Phillips E. Carosi G. Molina J. M. Workman C. Tomazic J. (2008). HLA-B*5701 screening for hypersensitivity to abacavir. N. Engl. J. Med. 358 (6), 568–579. 10.1056/NEJMoa0706135 18256392

[B36] Miyagawa F. Asada H. (2021). Current perspective regarding the immunopathogenesis of drug-induced hypersensitivity syndrome/drug reaction with eosinophilia and systemic symptoms (DIHS/DRESS). Int. J. Mol. Sci. 22 (4), 2147. 10.3390/ijms22042147 33670052 PMC7927043

[B37] Mockenhaupt M. Wang C. W. Hung S. I. Sekula P. Schmidt A. H. Pan R. Y. (2019). HLA-B*57:01 confers genetic susceptibility to carbamazepine-induced SJS/TEN in Europeans. Allergy 74 (11), 2227–2230. 10.1111/all.13821 30972788

[B38] Naranjo C. A. Busto U. Sellers E. M. Sandor P. RuIz I. Roberts E. A. (1981). A method for estimating the probability of adverse drug reactions. Clin. Pharmacol. Ther. 30 (2), 239–245. 10.1038/clpt.1981.154 7249508

[B39] Ng C. Y. Yeh Y. T. Wang C. W. Hung S. I. Yang C. H. Chang Y. C. (2016). Impact of the HLA-B(*)58:01 allele and renal impairment on allopurinol-induced cutaneous adverse reactions. J. Invest. Dermatol. 136 (7), 1373–1381. 10.1016/j.jid.2016.02.808 26996548

[B40] Pan R. Y. Chu M. T. Wang C. W. Lee Y. S. Lemonnier F. Michels A. W. (2019). Identification of drug-specific public TCR driving severe cutaneous adverse reactions. Nat. Commun. 10 (1), 3569. 10.1038/s41467-019-11396-2 31395875 PMC6687717

[B41] Pan R. Y. Dao R. L. Hung S. I. Chung W. H. (2017). Pharmacogenomic advances in the prediction and prevention of cutaneous idiosyncratic drug reactions. Clin. Pharmacol. Ther. 102 (1), 86–97. 10.1002/cpt.683 28295240

[B42] Rhoads J. L. W. Willson T. M. Sutton J. D. Spivak E. S. Samore M. H. Stevens V. W. (2021). Epidemiology, disposition, and treatment of ambulatory Veterans with skin and soft tissue infections. Clin. Infect. Dis. 72 (4), 675–681. 10.1093/cid/ciaa133 32047886

[B43] Rybak M. J. Abate B. J. Kang S. L. Ruffing M. J. Lerner S. A. Drusano G. L. (1999). Prospective evaluation of the effect of an aminoglycoside dosing regimen on rates of observed nephrotoxicity and ototoxicity. Antimicrob. Agents Chemother. 43 (7), 1549–1555. 10.1128/AAC.43.7.1549 10390201 PMC89322

[B44] Sassolas B. Haddad C. MockenhauptM. DunAnt A. Liss Y. BorK K. (2010). ALDEN, an algorithm for assessment of drug causality in stevens-johnson syndrome and toxic epidermal necrolysis: Comparison with case-control analysis. Clin. Pharmacol. Ther. 88 (1), 60–68. 10.1038/clpt.2009.252 20375998

[B45] Satapornpong P. Pratoomwun J. Rerknimitr P. Klaewsongkram J. Nakkam N. Rungrotmongkol T. (2021). HLA-B*13 :01 is a predictive marker of dapsone-induced severe cutaneous adverse reactions in Thai patients. Front. Immunol. 12, 661135. 10.3389/fimmu.2021.661135 34017337 PMC8130671

[B46] Su S. C. Mockenhaupt M. Wolkenstein P. Dunant A. Le Gouvello S. Chen C. B. (2017). Interleukin-15 is associated with severity and mortality in stevens-johnson syndrome/toxic epidermal necrolysis. J. Invest. Dermatol. 137 (5), 1065–1073. 10.1016/j.jid.2016.11.034 28011147

[B47] Suzuki Y. Kawasaki K. Sato Y. Tokimatsu I. Itoh H. Hiramatsu K. (2012). Is peak concentration needed in therapeutic drug monitoring of vancomycin? A pharmacokinetic-pharmacodynamic analysis in patients with methicillin-resistant staphylococcus aureus pneumonia. Chemotherapy 58 (4), 308–312. 10.1159/000343162 23147106

[B48] Symons N. L. Hobbes A. F. Leaver H. K. (1985). Anaphylactoid reactions to vancomycin during anaesthesia: Two clinical reports. Can. Anaesth. Soc. J. 32 (2), 178–181. 10.1007/BF03010047 3986655

[B49] Tamagawa-Mineoka R. Katoh N. Nara T. Nishimura Y. Yamamoto S. Kishimoto S. (2007). DRESS syndrome caused by teicoplanin and vancomycin, associated with reactivation of human herpesvirus-6. Int. J. Dermatol. 46 (6), 654–655. 10.1111/j.1365-4632.2007.03255.x 17550572

[B50] Tangamornsuksan W. Chaiyakunapruk N. Somkrua R. Lohitnavy M. Tassaneeyakul W. (2013). Relationship between the HLA-B*1502 allele and carbamazepine-induced stevens-johnson syndrome and toxic epidermal necrolysis: A systematic review and meta-analysis. JAMA Dermatol. 149 (9), 1025–1032. 10.1001/jamadermatol.2013.4114 23884208

[B51] Tassaneeyakul W. Jantararoungtong T. Chen P. Lin P. Y. Tiamkao S. Khunarkornsiri U. (2009). Strong association between HLA-B*5801 and allopurinol-induced Stevens-Johnson syndrome and toxic epidermal necrolysis in a Thai population. Pharmacogenet. Genomics 19 (9), 704–709. 10.1097/FPC.0b013e328330a3b8 19696695

[B52] Tassaneeyakul W. Tiamkao S. Jantararoungtong T. Chen P. Lin S. Y. Chen W. H. (2010). Association between HLA-B*1502 and carbamazepine-induced severe cutaneous adverse drug reactions in a Thai population. Epilepsia 51 (5), 926–930. 10.1111/j.1528-1167.2010.02533.x 20345939

[B53] Tohyama M. Hashimoto K. YasukawaM. Kimura H. Horikawa T. NaKajima K. (2007). Association of human herpesvirus 6 reactivation with the flaring and severity of drug-induced hypersensitivity syndrome. Br. J. Dermatol. 157 (5), 934–940. 10.1111/j.1365-2133.2007.08167.x 17854362

[B54] Tong S. Y. Davis J. S. Eichenberger E. Holland T. L. Fowler V. G. (2015). *Staphylococcus aureus* infections: Epidemiology, pathophysiology, clinical manifestations, and management. Clin. Microbiol. Rev. 28 (3), 603–661. 10.1128/CMR.00134-14 26016486 PMC4451395

[B55] Ushigome Y. Kano Y. Ishida T. Hirahara K. Shiohara T. (2013). Short- and long-term outcomes of 34 patients with drug-induced hypersensitivity syndrome in a single institution. J. Am. Acad. Dermatol. 68 (5), 721–728. 10.1016/j.jaad.2012.10.017 23182063

[B56] Vauthey L. Uckay I. Abrassart S. Bernard L. Assal M. Ferry T. (2008). Vancomycin-induced DRESS syndrome in a female patient. Pharmacology 82 (2), 138–141. 10.1159/000142729 18607115

[B57] Wang C. W. Chung W. H. Cheng Y. F. Ying N. W. Peck K. Chen Y. T. (2013). A new nucleic acid-based agent inhibits cytotoxic T lymphocyte-mediated immune disorders. J. Allergy Clin. Immunol. 132 (3), 713–722. 10.1016/j.jaci.2013.04.036 23791505

[B58] Wang C. W. Preclaro I. A. C. Lin W. H. Chung W. H. (2022). An updated review of genetic associations with severe adverse drug reactions: Translation and implementation of pharmacogenomic testing in clinical practice. Front. Pharmacol. 13, 886377. 10.3389/fphar.2022.886377 35548363 PMC9081981

[B59] Wang C. W. Tassaneeyakul W. Chen C. B. Chen W. T. Teng Y. C. Huang C. Y. (2021). Whole genome sequencing identifies genetic variants associated with co-trimoxazole hypersensitivity in Asians. J. Allergy Clin. Immunol. 147 (4), 1402–1412. 10.1016/j.jaci.2020.08.003 32791162

[B60] Weinborn M. Barbaud A. Truchetet F. Beurey P. Germain L. Cribier B. (2016). Histopathological study of six types of adverse cutaneous drug reactions using granulysin expression. Int. J. Dermatol. 55 (11), 1225–1233. 10.1111/ijd.13350 27421110

[B61] Yang S. C. Chen C. B. Lin M. Y. Zhang Z. Y. Jia X. Y. Huang M. (2021). Genetics of severe cutaneous adverse reactions. Front. Med. 8, 652091. 10.3389/fmed.2021.652091 PMC8319741 34336873

[B62] Yun J. Marcaida M. J. Eriksson K. K. Jamin H. Fontana S. Pichler W. J. (2014). Oxypurinol directly and immediately activates the drug-specific T cells via the preferential use of HLA-B*58:01. J. Immunol. 192 (7), 2984–2993. 10.4049/jimmunol.1302306 24591375

[B63] Yun J. Mattsson J. Schnyder K. Fontana S. Largiader C. R. Pichler W. J. (2013). Allopurinol hypersensitivity is primarily mediated by dose-dependent oxypurinol-specific T cell response. Clin. Exp. Allergy 43 (11), 1246–1255. 10.1111/cea.12184 24152157

[B64] Zhang F. R. Liu H. IrwAnto A. Fu X. A. Li Y. Yu G. Q. (2013). HLA-B*13:01 and the dapsone hypersensitivity syndrome. N. Engl. J. Med. 369 (17), 1620–1628. 10.1056/NEJMoa1213096 24152261

[B65] Zhao E. Y. Jones M. Jones S. J. M. (2019). Whole-genome sequencing in cancer. Cold Spring Harb. Perspect. Med. 9 (3), a034579. 10.1101/cshperspect.a034579 29844223 PMC6396343

